# Supplementary material for: Sustained Release of Tacrolimus Embedded in a Mixed Thermosensitive Hydrogel for Improving Functional Recovery of Injured Peripheral Nerves in Extremities
Source: Pharmaceutics. 2023 Feb 3;15(2):508. doi: 10.3390/pharmaceutics15020508 (PMC9960741; doi:10.3390/pharmaceutics15020508)
Supplement: Supplementary file 1 [file pharmaceutics-15-00508-s001.zip › pharmaceutics-2166696-supplementary.pdf]

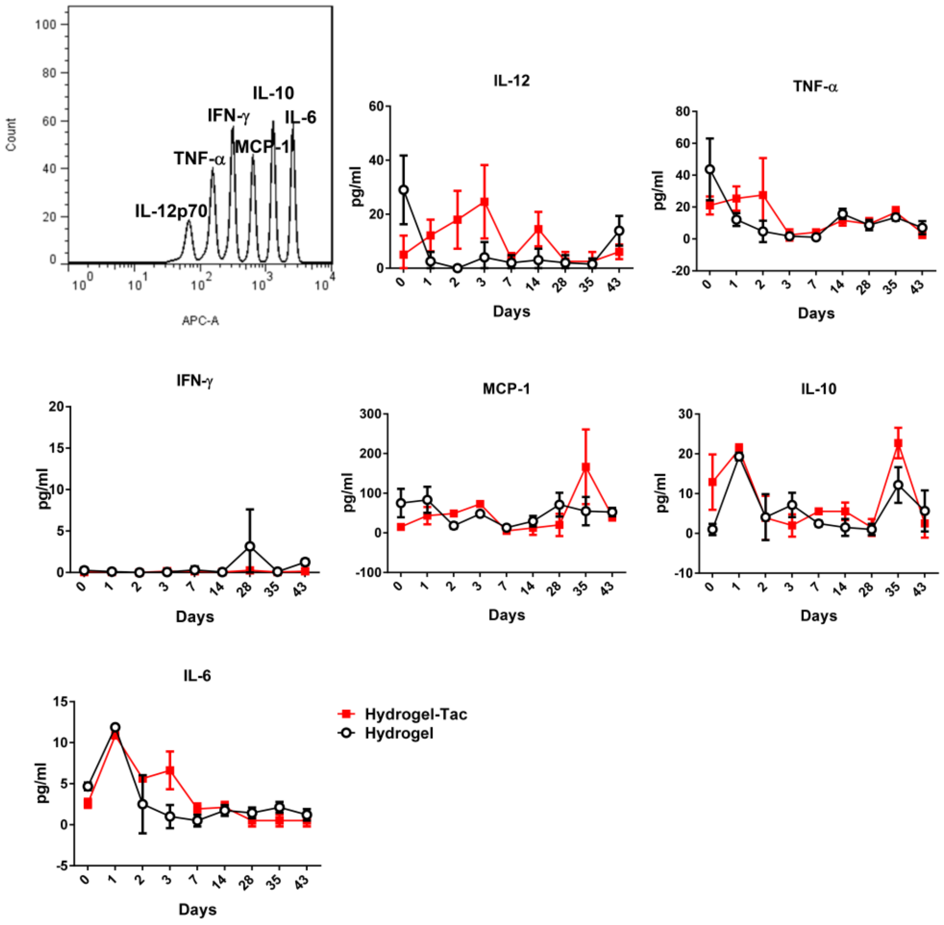

Supplementary Figure S1. Cytokine profiles in peripheral blood serum, assessed using cytometric bead array assay.

At different time points, blood serum samples were harvested from mice treated with one subcutaneous injection of 0.1 ml of hydrogel or Hydrogel-Tac (2 mg/ml). The concentrations of pro-inflammatory cytokines such as interleukin (IL)-12, IL-6, tumor necrosis factor-alpha (TNF-α, interferon (IFN)-γ, anti-inflammatory IL-10 cytokine, and chemokine monocyte chemoattractant protein-1 (MCP-1) were examined via cytometric bead array (CBA) assay using flow cytometry. The figure in the upper left displays various cytokine levels examined using flow cytometry. Various cytokine concentrations (pg/ml) were calculated according to the standard curve using FCAP Array multiplex analysis software. Each group had three mice. Mean ± SD was used to represent the statistical data.
